# Reasons to pursue a career in medicine: a qualitative study in Sierra Leone

**DOI:** 10.1186/s41256-017-0054-7

**Published:** 2017-12-04

**Authors:** Aniek Woodward, Suzanne Thomas, Mohamed Bella Jalloh, John Rees, Andrew Leather

**Affiliations:** 1 King’s Centre for Global Health and Health Partnerships, Division of Health & Social Care Research, Faculty of Life Sciences & Medicine, King’s College London, London, UK; 2 King’s Sierra Leone Partnership, King’s Centre for Global Health and Health Partnerships, Division of Health & Social Care Research, Faculty of Life Sciences & Medicine, King’s College London, London, UK; 3 College of Medicine and Allied Health Sciences, University of Sierra Leone, Freetown, Sierra Leone

**Keywords:** Undergraduate medical education, Medical students, Junior doctors, Reasons, Motivations, Selection criteria, Sierra Leone

## Abstract

**Background:**

Many low-income and crises-affected countries like Sierra Leone struggle with the recruitment and retention of their health professionals, particularly nurses and doctors. There are multiple factors that influence the ‘recruitment to retention’ pipeline. The first stage of an exploration into the issues influencing the availability of qualified health care workers may focus on the aspects which influence their entry into relevant educational programmes. This paper explores the reasons given by junior doctors in Sierra Leone for wanting to become a doctor. It also describes entry procedures into undergraduate medical education.

**Methods:**

In-depth interviews were conducted with purposively sampled junior doctors (*n* = 15) from the only medical school in Sierra Leone in October 2013. Digital diaries and two follow-up interviews were used to explore their evolving career experiences and aspirations until November 2016. In addition, semi-structured interviews with key informants (*n* = 20), including senior teaching staff at the medical school (*n* = 7), were conducted. Thematic analysis was used to explore linkages and themes across cases.

**Results:**

Six themes were identified. The most commonly mentioned reasons for wanting to become a doctor were a desire to help (theme 4) and the influence of family and friends, via role modelling (theme 2) and verbal encouragement (theme 3). Other motives were an interest from a young age (theme 1), being attracted by the job prospects (theme 5), and having an intellectual and science capacity (theme 6). Junior doctors gave at least two and up to six reasons for applying to enter the medical profession. Doctors were allowed entry to the medical school largely based on their previous academic performance.

**Conclusions:**

This study showed that multiple reasons underlie the decision to apply for entrance to medical school and the decision to enter medicine is complex. These findings may inform the review of future admission procedures by the medical school in Sierra Leone and similar settings, which is a crucial step in addressing the human resource needs for healthcare that currently exist.

## Background

Selection criteria and processes for entry into medical education, can be described as parts of the recruitment ‘pipeline’ of health workers [1]. The recruitment to retention ‘pipeline’ determines the number and type of health workers that will be available to serve a nation’s population.

Sierra Leone, like many low-income [2] and conflict-affected countries [3] struggles with the recruitment and retention of its health professionals, particularly nurses and doctors. One of the targets emphasised in the United Nations Sustainable Development Goals is the need to increase “recruitment, development, training and retention of the health workforce in developing countries, especially in least developed countries” [4]. As Sierra Leone ranks 181 out of 188 countries in the UN Human Development Index 2015 [5] it is considered one of those ‘least developed countries’ referred to in this goal.

Ratios of key professional groups to the general population are widely used to describe the strength of a health system. The latest available figures from the Ministry of Health and Sanitation (Government of Sierra Leone) is that there are 3 physicians, and 50 nurses and midwives per 100,000 population in Sierra Leone [6] as compared to the WHO recommended minimum level of 55 physicians, and 173 nurses and midwives per 100,000 [7]. Despite an increase in these ratios over recent years, current projections for Sierra Leone highlight a shortfall of 2800 healthcare workers in 2025 against the staffing requirements stated in the Basic Package of Essential Health Services [8]. This shortage of qualified healthcare staff in Sierra Leone is in part due to the civil war (1991–2002) and the recent Ebola crisis (2014–2016), which both led to the death and migration of many health workers. Additionally, a high percentage of doctors in Sierra Leone are working in administrative roles (37% of medical specialists) and a significant proportion of the healthcare workers are approaching retirement age (24% are over the age of 50 years). Although attrition rates have improved, loss of highly skilled staff to other sectors or other countries continues [8]. The need for increased education and recruitment of health personnel is recognised by the Government of Sierra Leone in the National Ebola Recovery Strategy (2015–2017), the Basic Package for Essential Health Services (2015–2020) and the Human Resources for Health Country Profile (2016).

Multiple factors influence the recruitment to retention pipeline including those related the educational sector [9]. Educational sector factors include the number and quality of graduates that are produced by educational institutions [9]. Attrition during education will affect the numbers of graduates available to enter the employment sector. Educational institutions rely upon the availability of individuals wishing to enrol into their programmes. Therefore, the first stage of any exploration into the issues influencing the availability of qualified health care workers should focus on the motivational factors which influence entry into relevant educational programmes.

Previous research has shown that ‘service to humanity’ is a common response for pursuing a career in medicine across a wide range of countries including Australia [10], Brazil [11], Finland [12], Ghana [13], Norway [14], Nigeria [15], South Africa [16], UK [17] and USA [18]. Some authors conclude that this is the most significant motivational factor [10, 16, 18] whereas others give this reason a lower ranking [15, 19].

Motivations often viewed as extrinsic in the literature include entering a high status profession that offers students the prospect of future respect and honour [11, 18], a sense of indispensability [20], financial gain [16], job security [16] and attractive career prospects [17].

Motivations generally considered as intrinsic in previous studies include personal interest [15], the desire for knowledge [18], interest in science and intellectual challenge [10, 17, 21] and the perception of medicine being a good academic choice [16].

A variety of background factors such as gender [10, 12, 14, 17, 20], socioeconomic status [22], personality [22], academic achievement [20, 23], having medical family members [20], which could influence motivation to choose medicine or a particular medical speciality have also been considered.

The question of how motivation to study medicine might vary across different countries or cultures remains largely unanswered although one study comparing medical students in Ghana and UK suggests they might be similar [24]. Investigating the motivations of young people applying to enter undergraduate medical educational programmes have mostly originated from high resource settings and few are from the low resource setting or more specifically from Sub-Saharan Africa.

This paper explores the reasons given by junior doctors in Sierra Leone to start undergraduate medical education. Through a variety of qualitative methods we tested the relevance of factors, including those identified in previous studies, in the context of Sierra Leone. Gaining a better insight into the motivations to study medicine in Sierra Leone is an important first step to addressing the significant lack of qualified and specialist doctors that are vital for the country’s healthcare system.

## Methods

### Research design

This paper is part of a wider qualitative longitudinal (QL) interview study on the career experiences and aspirations of junior doctors in Sierra Leone.

### Recruitment of participants

Junior doctors were purposively selected based on maximum variation (mixture of men/women, years of graduation and career stages (house officer, medical officer, resident, various work/study environments). This type of sampling, which originated from grounded theory, looks for “information-rich” cases [25]. Doctors who graduated from the College of Medicine and Allied Health Sciences (COMAHS), which is the only institution that educates medical doctors in Sierra Leone, from 2002 onwards (i.e. post-civil war) were eligible for participation in the study.

Personal contacts via the King’s Sierra Leone Partnership (KSLP) were accessed firstly to identify potential junior doctors for participation, followed by snowballing, which involved asking participants to recommend other participants [26]. KSLP is a collaboration between King’s College London, the Centre for Global Health & Health Partnerships, and institutions in Sierra Leone that helps to strengthen the health system [27]. This generated a list of contact and background details of 48 junior doctors. Those with characteristics needed to achieve maximum variation were asked by the lead researcher (AW) to participate by phone or email. The aim was to achieve a sample size of about 15. This size was chosen to allow for a variation of doctors to be included while still being feasible within restrictions of the project. In total 28 doctors were approached and 15 agreed to participate. Not answering the phone or non-response via email after initial approach was the main reason for not being interviewed.

Secondly, key informants were purposively sampled to get a range of perspectives on advancements and challenges in medical education and workforce development post-conflict (e.g. senior teaching staff at the medical school, policy makers, NGO workers involved with programmes that train health workers, medical students), using a similar recruitment strategy to that used with junior doctors. A list of 26 key informants were identified by AW, 22 were invited and 20 agreed to participate. Two refused because they did not have time to be interviewed.

### Methods of data gathering

Five different methods were applied as shown in Table 1. A life-history approach was employed via in-depth interviews with junior doctors. This approach is understood as personal narratives situated in time and context, and was found applicable in a recent publication about career experiences of health workers in post-conflict settings [35, 36].

**Table 1:** Overview of methods applied for larger qualitative longitudinal study

Method	Objective	Approach	Timeline
*1) Semistructured interviews with key informants*	To describe the policy landscape on medical training	A purposively selected sample of key informants (*n* = 20) was interviewed face-toface, including senior teaching staff (*n* = 7). A topic guide^a^ structured interview was used including two group interviews.	Oct ‘13; interviews lasted 27–140 min (63 min average)
*2) Document analysis*		Relevant policy documentation and statistics were obtained (if available and accessible) via web searches and contacts.	Oct ‘13 to Sept ‘17
*3) In-depth interviews with junior doctors*	To explore career experiences and aspirations up to that time	Purposively selected sample of doctors (*n* = 15) was interviewed. Participants were asked to complete a lifeline chart^b^ at the start of the interview to aid reflection on past experiences. Completed charts and a topic guide^c^ helped structure individual interviews. Fourteen were conducted face-to-face and 1 via Skype.	Oct ‘13; interviews lasted 67–126 min (86 min average)
*4) Digital diaries*	To explore evolving career narratives and aspirations	Participants were asked to record (via email, sms or WhatsApp) accounts of ‘critical events’ related to their career. A guidance sheet was developed to facilitate recordings and emails were sent to invite recordings 4 times per year. 46 digital diaries were collected.	Feb ‘14 to Nov ‘16
*5) Follow-up interviews with junior doctors*		Follow-up interviews with previously recruited doctors. First interviews focused on experiences related to the Ebola crisis and the second on career aspirations. Eight of 15 junior doctors were initially interviewed via Skype and 7 for the second follow-interviews (6 via Skype; 1 face-to-face).	May ‘15 (interviews lasted 27 min average); Nov ‘16 (interviews lasted 30 min average)

^a^Topic guides for interviews with key informants were based on the literature [28–31] and experiential knowledge. Guides were adapted ^a^ for different types of key informants but generally covered the following topics: evolution of medical school policy; impact of civil war on human resources for health and medical education; coordination, monitoring and regulation of COMAHS; planning and recruitment of medical workforce; quality of medical education; postgraduate medical education and professional development opportunities; financing of medical school; migration/retention of doctors; deployment and distribution of doctors; and gender issues; ^b^Lifeline chart recorded key family events (births, deaths, illnesses), places lived, educational and employment history; ^c^Topic guide for initial interviews with junior doctors was based on the literature [32–34] and experiential knowledge and covered the following topics: reasons to start medical education; experiences and financing of medical school; work experiences and options since graduation; attitudes to migration, attitudes to public and private sector work; quality control and regulation; job market and career aspirations; social demands and expectations; and gender issues

### Interviews

A pilot small focus group was conducted with three medical students from Sierra Leone to test the life-line chart and part of the interview guide for junior doctors. No changes to the format of the interview were necessary as students understood all questions. The interview guide for key informants was piloted with an NGO worker. The place and time of each audio-recorded interview was negotiated with each participant and the researcher. All participants were provided with an information sheet and then gave written informed consent prior to the interviews. Most interviews were conducted face-to-face during a fieldtrip to Freetown in 2013. Follow-up interviews with junior doctors were mostly carried out remotely via Skype or telephone to minimise risks related to fieldwork (the Ebola crisis happened during the study period).

### Data analysis

All interviews were transcribed and analysed with NVivo 10.2.2. © QSR International (a qualitative computer software programme) by the lead researcher (AW). Thematic analysis was applied to explore relationships and ‘themes’ across the interviews [37]. After familiarisation with the transcripts, data was initially coded and further refined, ordered, reordered, categorised and themed until a final coding framework was identified. This matrix was then applied to all transcripts.

Various recommended approaches [38] were considered to increase validation of the interpreted data. Firstly, through constant comparison, differences and similarities were explored. Differences by gender and cohort year were the main foci. Efforts were made to search for ‘deviant cases’, which are those that might disconfirm emerging themes [25]. Secondly, single counting was used to indicate how strong the evidence of certain experiences was amongst the study cohort [39]. Lastly, initial findings were discussed with co-authors (ST and MBJ) to check if results were aligned with the study objectives.

The results section includes numerous direct accounts, which is particularly important in the life-history approach [40]. The source of each quote cited is indicated as junior doctor (JD) or senior teaching staff (TS), accompanied by a randomly assigned number for each participant, to illustrate the variety of responses. Background information such as gender or cohort of JD is provided where relevant.

## Results

### Participant characteristics

Table 2 provides an overview of the characteristics of all study participants analysed for this paper. Only senior teaching staff were included as part of the key informant sample, because they were the only ones to comment on some of the topics described in the results. Findings from other key informants will be used in other analyses.

**Table 2:** Study participant characteristics as per November 2013 (unless otherwise specified)

Sample	Characteristic	
*Junior doctors (JD) (n* = *15)*	Age	29 years (average); 24–35 years (range)
	Sex	60% male; 40% female
	Graduation year^a^	2015 (*n* = 1), 2013 (*n* = 2), 2012 (*n* = 4), 2011 (*n* = 2), 2010 (*n* = 1), 2009 (*n* = 3), 2008 (*n* = 1), 2006 (*n* = 1)
	Children^a^	yes (33%); no (67%)
	Marital status^a^	single/dating/divorced (60%); married (40%)
	Religion	53% Christian; 47% Muslim
*Senior teaching staff* ^*b*^ *(TS) (n* = *7)*	Sex	72% male; 28% female

^a^Characteristic as per November 2016;^b^Includes those formally employed by COMAHS and those teaching on a voluntary basis

### Making the ‘decision’ to pursue medicine: an interplay of various reasons

Six themes or reasons to pursue medicine were identified. Junior doctors gave at least two and up to six reasons for applying to enter undergraduate medical education.

#### 1. A child’s dream: interested at a young age

Two junior doctors (2/15) recalled always wanting to become a doctor:

“It [becoming a doctor] has always been my dream. I have been going through kindergarten…if I grow up I would like to be a doctor.” (JD2)

Although not everyone could pinpoint the exact time when they were set to study medicine, for most (8/15) this desire started somewhere during primary school:

“I think when I was six or seven [when I wanted to become a doctor].” (JD5)

Others (5/15) remembered considering medicine during secondary school:

“ At sixth form, there were four in our class who always wanted to become a doctor.” (JD8)

Becoming a doctor, however, was not always the first aspiration. Five doctors (5/15) described initially wanting to do: “something mechanical” (JD7), “physics and maths” (JD13), teaching (JD9), chemistry, (JD4), and arts and humanities (JD14). Several (4/15) contemplated opting for engineering:

“Initially I had two options. Being an engineer and a medical doctor.” (JD3)

One doctor explained deciding against engineering “because I was afraid of too much mathematics and drawing” (JD1).

Junior doctors were influenced by their family and friends in two ways: role modelling (theme 2) and verbal encouragement (theme 3).

#### 2. The familiar path: inspired by role models in health professions

A third of doctors (5/15) were inspired by one or more family members in the health professions:

“I had an uncle when I was like growing up. He was like my hero, he was a cardiologist, he’s dead now. And I always wanted to be like him.” (JD15)

Having family members in the health professions increased exposure to the work of health professionals and patient care:

“When I came to Freetown during my primary school years, at first I was living with my father’s younger brother, whose wife was a nurse. During the week, we would visit Connaught hospital more than three times. So each time I went to the hospital and see people and see doctors and nurses, I would stand there and admire them.” (JD10)

One female doctor (1/6) learnt from her aunt, who was a nurse, that women could become doctors just like men:

“So then when I was eight, nine, ten it was very rare to get a female doctor. Especially one that lived in the country because most were maybe out of the country or into public health maybe. So it was very rare. I only learnt from my aunt [a nurse] and I was shocked that women can also become doctors.” (JD6)

Two junior doctors were inspired by family members who were male doctors and three by family members who were female nurses. Both male and female doctors were inspired by male and female role models.

The influence of relatives and friends by means of verbal encouragement was a related theme.

#### 3. Being ‘talked into it’: influence of family and friends

Half of sampled doctors (7/15) were advised by one or more family members to pursue a career in medicine:

“But after my A-levels I still couldn’t decide where to go [for further education]. And my mum liked medicine so she told me ‘just go for medicine’. So I came into medicine.” (JD11)

Parents were most commonly cited (*n* = 5) to influence their career decision-making, followed by siblings (*n* = 2) and uncles and aunts (*n* = 2). While most (14/15) did not elaborate on why their families wanted them to become a doctor, one junior doctor, explained being groomed by her father to pursue medicine to fulfil his dream:

“Do you know what, my dad always wanted to be a doctor… So it was his dream. He wanted to be a doctor. So he said ‘look one of my kids is going to be a doctor’. So every member of my family went through the science stream.” (JD14)

And another thought her parents just wanted her to be called a doctor:

“But being a medical doctor now I was encouraged by my parents to choose medicine…They want me to be called a doctor.” (JD3)

Besides family, two doctors were influenced by friends. One had friends who were already in the medical profession:

“And some [doctors] were my friends. Yeah. The doctors by then they were encouraging me [to enter medical education].” (JD5)

And another had friends in medical school:

“So if you have a friend who is older than you and is in medical school. Because they were not doctors by then, they were just in medical school. So they said ‘come to medical school, it’s fine here’.” (JD12)

Besides the influence of friends and family, another commonly mentioned reason given is that of an innate desire to want to help.

#### 4. Feeling called to relieve suffering: the doctor as healer

Two thirds of (10/15) junior doctors gave the relief of suffering as the reason for entering the medical profession. Most (7/15) expressed this as a longing to help people in general:

“It’s just that I had this urge of serving people” (JD2)

One doctor wanted to ensure his family was looked after when his uncles, who are senior doctors, come to the end of their careers:

“If they [uncles who are doctors] go away, who is going to replace them and give my family the opportunity to have someone to take care of them when they’re ill? So that served as an inspiration to like commit [to become a doctor].” (JD12)

Two doctors (2/15) added that they aspired to improve their country and health system:

“We have a long way to go in this country when it comes to health and education. So I always wanted to help in that part.” (JD15).

For another two (2/15), the death of their fathers seemed to be decisive moment in their career planning:

“And then in 1999 when my father died I was like ‘oh maybe if I become a doctor I’ll be able, you know, stop people from dying’. And that was what I was thinking: if I become a doctor I would be able to help more people. Maybe I would be able to stop and know what is wrong with my father and things like that. So there and then I started looking at the medical field.” (JD10)

One more theme involved the job prospects.

#### 5. Attracted by the prospects of the job

A handful of junior doctors (5/15) were attracted by the job prospects of a career in medicine. Especially the respect of the profession in society seemed a pull factor (3/15). One doctor explained this high esteem might have been due to lack of medical doctors at the time:

“I think I held doctors at a very high esteem. They are always straight-headed [laughs]. I think at the time, it was difficult to come by a doctor. So every time you meet a doctor; you know he is a big man, a career.” (JD8)

Another doctor (1/15) was attracted by the possibility to perform multiple roles:

“The reason why I chose medicine was because it can be both professional and it could be academic. So I can be an academic here in the medical field. I can be a lecturer, I can be a Dean, I can be part of the University as well.” (JD12)

And job security was another reason given for choosing medicine (1/15):

“The other thing also that pushed me into [medicine]…was being secured of having an employment when I finish.” (JD7)

A final theme for starting medical education related to having an intellectual and science capacity.

#### 6. Having the brains: intellectual and science capacity

Having the necessary intelligence was another reason why some (5/15) considered medicine:

“I was a bright student.” (JD6)

Besides being bright, an aptitude for science subjects (i.e. physics, chemistry and biology) seemed important in their career decision-making as this was raised by three doctors (3/15), including:

“And throughout secondary school I was really good in chemistry and biology. And I would say ‘oh let me just go for this’. So by the end of the day I enrolled into medicine.”(JD10)

Once these young people had decided to apply for medical education, they had to be selected by the medical school.

### Enrolment into medicine in Sierra Leone: entry requirements and process

COMAHS, part of the University of Sierra Leone, is the country’s only medical school. This part of the results describes the enrolment process and requirements into COMAHS and is mainly based on the views of junior doctors (*n* = 7) who accepted to be re-interviewed in November 2016. Insights of interviewed teaching staff (*n* = 7) at the medical school are included where possible.

#### Entry requirements: focus on previous academic performance

There was consensus amongst accounts by junior doctors that selection relied largely on secondary school grades for English language, mathematics and three science subjects (physics, chemistry and biology). Applicants were required to submit their expected grades along with the application form to the College to show their interest in studying medicine. If they fulfilled the entry requirements set by the University of Sierra Leone (West Africa Senior Secondary School Certificate Examination (WASSCE) credits of C5 or better), they were shortlisted and called for an interview with a panel of lecturers at COMAHS.

“We [applicants] obtained the application form from the University [of Sierra Leone]. You [applicant] fill in the application form and you attach your WASSCE. So the requirement is that you’ll have five or better credits in maths, physics, chemistry, biology and English language. Five subjects. If you have at least a credit in those subjects then you’ll be admitted into the preliminary class [of medical school].” (JD12)

“First of all we [applicants] were shortlisted based on our [secondary school] results. And after shortlisting you were invited for an interview.” (JD14)

During the interview, junior doctors recalled being asked by the interview panel – consisting of lecturers at COMAHS – about their motivation to study medicine (5/7) their family and geographical background (2/7) and financial ability to pay for medical education (2/7):

“Why do you want to do medicine? Your background; where you’re from?... And of course things like if you can afford the cost [related to studying medicine] that was also part of the interview questions.” (JD8)

Four junior doctors (4/7) explained the interview was also used by the panel to verify applicants’ secondary school grades:

“During the interview, we [applicants] had to prove whether the results we submitted [during the application for medical school] meet the [secondary school] grades we actually got. Because we applied [to medical school] before the [secondary school] results were published.” (JD3)

A senior staff member at COMAHS (TS4) indicated they plan to computerise applications so that authenticity is checked and confirmed prior to future interviews.

Three out of four junior doctors who were prompted about whether connections (having friends or family within the College or the health profession) had an influence on selection into medical school, had heard rumours about this happening. None of those prompted, however, experienced this first hand.

“I had nobody in the extended family who was part of the University. So I was a nobody. But I entered the university. I went through, I sailed through…But I know that is a widely held opinion, because some people think, or believe that for some of our colleagues who had relatives on the examination [board] or whose parents were lecturers in certain departments, they sailed through [medical school] with less challenges…But I personally would like to believe that was just a matter of coincidence.” (JD13)

It was unclear whether interviews were standardised as one doctor commented:

“It depends on the [interview] panel what they choose to ask you.” (JD5)

No testing of intellectual capacity was made during the interview:

“No there’s no [intellectual] test. Just your WACCSE results and then the interview.” (JD12)

Although a senior COMAHS staff member (TS4) commented “the University wants to introduce university entrance exams” to be able to select the best students in the future.

Junior doctors (2/7) remarked most applicants invited for an interview were admitted to COMAHS:

“Almost 90% of those people who are invited for an interview are subsequently admitted.” (JD12)

Grades determined at which year a student would start his or her education: those with lower grades, calculated via an aggregate system at the College, entered at premedical year one (or ‘preliminary’ prior to 2002) and were required to take 2 years of basic science programmes before starting the MB ChB programme. Those with higher grades entered at premedical year two and were required to complete 1 year of basic sciences before progressing to the MB ChB course.

“Then after the interview they [those involved in shortlisting of applicants at COMAHS] did a second shortlist and if your name was on the shortlist then automatically it was decided whether you would go into the preliminary year [of Medical School], based on your results, or if you could go straight into first year.” (JD14)

Due to the “system of education” (JD3) in Sierra Leone, however, most applicants did WASSCE instead of A-level exams. This meant they had to go through premedical education first before entering the Bachelor of Medicine and Bachelor of Surgery (MB ChB) programme. The few who immediately entered first year of the MB ChB programme were those with “the required grades” (JD5) of an A-level or previous undergraduate science degree. In the sample of 15 junior doctors interviewed for this study: one had direct entry into the MB ChB programme, eight did 1 year and six did 2 years of premedical education.

A student’s average for their final exam of premedical education determined whether a student would enter first year of medicine or pharmacy:

“So in premed two you have to pass all of your sciences and then your average must be 65% and above to be admitted to do medicine” (JD8)

Accounts indicate that the number of medical students increased since the end of Sierra Leone’s civil war in 2002. Results also suggest an additional year of premedical education was introduced during that time.

#### Post-conflict: increased number of medical students and additional year of premedical education

Senior teaching staff and junior doctors alike experienced a consistent growth in the number of entrants at COMAHS in the last two decades.

“Well in those days [during the war] we had about 12-13 [medical students]. It increased to about 20 in early 2000. And now [2013] it is increased to an average of 45 students.” (TS3)

“I was in a class of just 19 [in 1998] and now [in 2013] the classes are 40 to 50.” (JD14)

Interviews with junior doctors also indicate that a second year of premedical education was added post-conflict.

“At first it [premedical education] used to be just one year, one prelim [short for ‘preliminary’], but in 2002, I think because of the population of students applying, so if you don’t have particular grades like in physics or chemistry…they [senior staff at the Medical School] would ask you to go to premed one.” (JD5)

## Discussion

These results show multiple reasons underlie the decision to apply for medical education which is in line with findings from a qualitative study on medical students in South Africa [16]. In addition, it is supportive of previous research using questionnaires that rank medical student motivations, which show students have a variety of motivations [14, 17, 20].

This study also shows that the decision to enter medicine is a process rather than a single point in time decision. For some doctors this innate desire to become a doctor has been there as long as they can remember, while for others the desire can be traced back to primary school age when they felt inspired by a family member in a health profession, and for others this career option became more definite towards the end of secondary school when they realised they enjoyed and/or had an aptitude for science subjects. More often it is difficult to pinpoint *when* and *what* reasons come into play and *how* these different reasons may have interacted with one another so that a junior person became sufficiently motivated to apply for medical school. Longitudinal studies, which explore the career ambitions of young people through primary and secondary school, are needed to answer such questions.

The influence of family and friends, whether by inspiration, encouragement or pressure, was particularly important in this study. Other studies conducted in sub-Saharan Africa had similar findings [16, 41, 42]. Whether family influence is more important in choosing medicine in low income countries compared to high income countries requires further exploration. The decision to enter medicine for junior doctors in this study was largely made during the civil war (1991–2002). During this time the country’s health, education, and socio-economic systems were unstable, meaning young people were particularly reliant on family support.

Family and friends in the health professions served as role models in this study. Although there are different definitions of ‘role models’ and ‘role modelling’ in the medical education research literature, generally a role model is seen as a person who is considered worthy of imitation because of their social role, status, or personal and/or professional qualities [43]. A previous study found that gender-matching of role models is more important for female than for male college students [44]. Results from this study show that medicine was a gendered profession at the time these junior doctors made their decision to enter medicine; doctors were predominantly male while nurses were largely female. The number of female medical students in Sierra Leone has, however, significantly increased in recent years, with cohorts even reported as gender balanced. Whether this is due to an increase in female medical graduates and therefore the availability of female role models, or due to changes in local beliefs about the roles of women in society, or any other reasons, needs further exploration. A gender breakdown of health workers in 2016 indicates women remain underrepresented amongst medical officers (26.7% female vs. 73.3% male) and medical specialists (5.4% female vs. 94.6% male) in Sierra Leone [8].

This also raises the possibility that many students will be receptive to continued role modelling by the academic staff and healthcare professionals that they encounter during their course. For those students who do not come from backgrounds with medical connections, the absence of role models might mean that they are at a disadvantage in terms of considering medicine or knowing what to expect of the medical degree course. Targeted outreach to secondary school students and development of student support services might be considered in order to address this issue and widen access to the course.

None of the junior doctors in this study mentioned potential financial gain as a reason to enter medical education, which contradicts previous research [15–18]. They did mention job prospects and security, which might be regarded as more socially acceptable ways to express the practical issue of financial reward. The lack of mention of financial rewards, at least as initial motivation to enter medicine, might also suggest that non-financial incentives might be as, or even more, important than financial ones in motivating junior doctors. This is in line with findings from a systematic review on motivation and retention of health workers in developing countries, which concluded that financial incentives are insufficient to motivate health workers [45]. A questionnaire which allows medical students or graduates in Sierra Leone to choose from, and possibly rank, various non-financial and financial motivations is needed to further investigate the importance of such motivators. Such a questionnaire should preferably be repeated at various points throughout their careers to capture changes over time.

These results could also be helpful when developing and delivering the medical curriculum. Comparison of students’ motivations to this desired outcome can help highlight where students are most and least aligned to the expectations of the institution. For example, the motivation described as ‘feeling of being called to relieve suffering’ might suggest that these students will be inclined to focus on patients and expect that of their medical education. However, if their early years of education do not include exposure to patients this motivation will not match their experience and could lead to disillusionment, reduced engagement and withdrawal from education. Studies have shown that idealism amongst medical students can decline within the early years of their course and an influential factor in this change in motivation is the interaction students have with medical staff [46].

Previous academic achievement is the dominant factor that determines an applicant’s entry into medical education in Sierra Leone. While previous academic performance can predict the outcome of a medical career [21, 23], there is consensus in the literature that this not a ‘perfect’ predictor for future performance. One recent review concluded that there is a lack of “evidence that medical school applicants with higher grades go on to become better physicians” [47]. Other non-academic attributes are, therefore, important to assess as part of the selection process.

Results indicate that academic records at the medical school in Sierra Leone are used as a method to shortlist from a pool of applicants and also as a final selection method. As the pressure to train more doctors is likely to remain, COMAHS will continue to face challenges in terms of ensuring that students entering the programme are of a suitable academic standard. One aspect to be considered in the admissions process is the role of the interview. If one of the aims is to assess the suitability of students for undertaking the course and a future career in medicine, then consideration of tests that explore students’ approach to learning e.g. ability to problem solve or work in a team could be of additional value.

When junior doctors in this study entered undergraduate medical education (1998–2008), the interview was focused on checking the authenticity of secondary school grades rather than exploring the non-academic attributes of interviewees. This might have been important at the time with secondary school results being unreliable during the conflict and immediate post-conflict period. It remains unclear what attributes the medical school was and is looking for in its applicants. The definition of a ‘successful interview’ is not clarified in the latest curriculum [48]. Evidence suggests that multiple mini-interviews might offer more reliability and validity than traditional interviews, although their impact in terms of equity and performance of applicants needs to be further researched [47]. Other selection methods could also be considered, such as aptitude or situational judgement tests. The need for more studies to determine the most effective combination of selection tools [47] and variables [49], has been previously raised. Medical schools in countries like Sierra Leone would particularly benefit from evidence on best practices from low-income countries, where human and financial resources of medical schools are more constrained and, therefore, some selection tools might not be feasible nor desirable to implement.

Rumours of favouritism in the selection process were prevalent at the time junior doctors in this study entered medical education (between 1998 and 2008). Having an even more robust and transparent selection system in place might combat such perceptions of unfairness. To aspire to fairness and inclusivity is also important in creating a people-centred health system. For example, medical schools in South Africa aim to improve “inclusivity among the students selected in order to meet the needs of the diverse socioeconomic and cultural populations that qualifying doctors will serve in [the] future” (p80) [50].

One of the objectives in the Human Resources for Health Strategy for Sierra Leone is to “plan and implement interventions in pre-service education to increase the number of health workers in areas where addressing shortages is critical to meeting the country’s health needs” (p32) [6]. This will include creating a long term national education plan which will consider staffing needs and the capacity of health education institutions to increase production of targeted health care professionals. An increase in medical graduates is likely to be needed. The addition of an extra year of premedical education was introduced to get those with lower secondary school grades up to the required entry into medicine and this meant that there was a larger pool of secondary school graduates to choose from during a critical period for Sierra Leone. However, if a further increase in entrants is needed, attention will need to focus on strengthening the teaching of science subjects at primary and secondary level. This will require support from the Ministry of Education.

### Study limitations

This study has several limitations that need to be taken into consideration. Firstly, this study only includes the views and experiences of junior doctors who successfully went through medical education in Sierra Leone. This means results cannot be compared to those who:

- Considered medicine but eventually went into another profession (engineering, arts, chemistry, maths were other studies participants considered);
- Failed the initial selection process;
- Were selected but not able to start education because they were not able to afford it or for any other reason;
- Entered education with the aim to become a doctor but the exam at the end of premedical education decided the student moved to pharmacy instead of medicine;
- Did not finish medical education;
- Did their undergraduate medical education abroad.

These people not included may give different reasons for wanting to become a doctor, and/or may come from a different background than the junior doctors who participated in this study. For example, those who study abroad might come from more affluent families as medical education abroad is generally more expensive. Future research, therefore, should consider including some of these groups and explore possible differences between them.

Secondly, results are based on the views of a limited and purposively selected sample. This means there is potential selection bias and consequently results may not be generalisable to the entire medical student population at COMAHS.

Thirdly, there may be recall bias as the decision to become a doctor was made many years before the interviews. During the interviews, however, participants easily gave their reasons for wanting to pursue a medical career.

Fourthly, data on entry requirements was based on a reduced sample of junior doctors (7 of 15). Views on this topic were mainly explored in final follow-up interviews (as this topic became of interest after data analysis). Some participants did not respond to the request to be re-interviewed, which means their views could not be included. Despite this, data seemed saturated as male and female doctors and those from different cohorts gave similar answers.

Fifthly, the perspectives of senior teaching staff were under-represented in the results. The main reason is that interviews with senior teaching staff focused on their views with regards to policy making, quality, and financing of medical education, job market for and deployment of medical doctors. They were not asked about the reasons why medical students at COMAHS entered medicine. While entry requirements were part of the topic guide for staff working at COMAHS, because most participants were time constrained, not everyone was asked about this subject. Future research should consider exploring their views in more detail.

## Conclusions

This study provides an insight into what motivated junior doctors to start undergraduate medical education in Sierra Leone as well as how they were selected. Findings on motivations and entry requirements may inform the review of future admission procedures in Sierra Leone and similar settings, although the qualitative nature and small sample of this study requires caution in generalising findings. The paucity of evidence, particularly for resource-constrained countries, means more research is urgently needed to help those responsible for medical workforce planning and education to make informed decisions.

## Abbreviations

COMAHS: College of Medicine and Allied Health Sciences
JD: Junior doctor
KSLP: King’s Sierra Leone Partnership
MBChB: Bachelor of Medicine and Bachelor of Surgery
QL: Qualitative longitudinal
TS: Senior teaching staff
WASSCE: West Africa Senior Secondary School Certificate Examination
